# Profile susceptibility to fluconazole and voriconazole antifungals by species of *Candida albicans *isolated from urine culture

**DOI:** 10.1186/1753-6561-8-S4-P34

**Published:** 2014-10-01

**Authors:** Laura Wiebusch, Danielle Lonchiati, Luana Rodrigues, Camila Dantas, Adriana Almeida, Kelly Oliveira

**Affiliations:** 1 Universidade Federal da Grande Dourados, Dourados, Brasil

## Background

The term candiduria can be defined as the observation of yeasts on direct examination of urine through the presence of pseudo-hyphae and fungal growth in urine culture [1]. The yeast Candiduria can be detected in the urine in patients who have bladder colonization and in patients who have urinary tract infection [2]. Currently, there are few options in the medicine antifungal market for the treatment of urinary tract infections. And the most used are amphotericin B, ketoconazole, fluconazole and voriconazole. Therefore, the determination of the profile of antifungal susceptibility among isolates of *C. albicans *urine in patients, it is so important as epidemiological marker that serves to guide therapeutic procedures [3]. Thus, many methods have been tested as alternative to the antifungal susceptibility, as the method of agar disk diffusion M44-A12. So, this study aimed to evaluate the antifungal susceptibility profile of *Candida albicans *isolated from the urine of patients admitted at the university hospital in Dourados - MS.

## Methods

In this study we used 24 samples of *Candida albicans *from urine cultures of patients hospitalized at the university hospital of Grande Dourados, from June 2010 to June 2012. The yeasts were isolated and identified according to the conventional method. The antifungal susceptibility of isolates of *Candida albicans *was evaluated using the disk-agar diffusion method, according to the standards of the Clinical and Laboratory Standards Institute (CLSI) M-44 [4], using as antifungal fluconazole and voriconazole. The tests were performed in duplicate and the reading of the plates was performed using the methodology described by Demitto, 2012 [5].

## Results and conclusions

The results obtained in testing susceptibility to the antifungal fluconazole showed that 54.16% of the samples were sensitive and 45.83% were resistant to the same drug. The antifungal voriconazole showed that 54.16% of strains were sensitive and 45.83% were resistance to the same drug. According to the results obtained by Demito et al. 2012, the antibiotics fluconazole and voriconazole showed equivalent efficacy in vitro. So, this maybe can be related to cross-resistance due to the similarity of the chemical structure of these azoles, because strains resistant to fluconazole also showed resistance to voriconazole.
